# Current and Future Trends in Life Sciences Training: Questionnaire Study

**DOI:** 10.2196/15877

**Published:** 2020-04-24

**Authors:** William Magagna, Nicole Wang, Kyle Peck

**Affiliations:** 1 Siemens Healthineers Newark, DE United States; 2 Department of Learning Performance System Pennsylvania State University University Park, PA United States

**Keywords:** professional training, training with technologies, life sciences professionals, mixed methods

## Abstract

**Background:**

Every year, the life science field spends billions of dollars on educational activities worldwide. The continuing professional development of employees, especially in this field, encompasses great challenges. Emerging technologies appear to offer opportunity, but relatively little research has been done on the effectiveness of pedagogies and tools that have been used in the life sciences, and even less research has been devoted to understanding the potential power of emerging options that might determine the field’s future.

**Objective:**

In collaboration with the Life Sciences Trainers & Educators Network (LTEN), this study investigated the current state of the pedagogies and tools currently adopted by corporate training professionals in the life sciences as well as the professionals’ perceptions of the impacts of emerging technologies on training.

**Methods:**

This study adopted a mixed methods approach that included a survey and a follow-up interview. The survey consists of 18 broad questions with 15 subquestions in each of the five specific sectors of the life sciences field. Interviews were conducted by phone and lasted approximately 40 minutes, covering 18 questions designed to follow-up on findings from the survey items.

**Results:**

Both survey and interview results indicated that the professionals were not satisfied with the status quo and that training and education in this field need to change. Most of the techniques and tools currently used have been used for some time. The professionals surveyed were not satisfied with the current techniques and tools and did not find them cost-effective. In addition, the respondents pictured the future of training in this field to be more engaging and effective.

**Conclusions:**

This is the first study in a series designed to better understand education and training in the life sciences on a macro level, in order to build a foundation for progress and evolution of the future landscape. Next steps involve developing strategies for how to extend this vision throughout individual organizations.

## Introduction

### Background

The life sciences have been evolving at a staggering rate in every aspect. According to Kaufman [1], “Medicine has gone through major changes over the last 50 years. Today it is recognized that medical knowledge doubles every 6-8 years, with new medical procedures emerging every day.” In gene ontology, there are about 250 ontologies accessible to professionals in the field [2]. In addition, new services, technologies, and applications have emerged through the evolution of life sciences education [3,4]. For instance, studies have suggested the use of artificial intelligence in improving medical imaging [5,6] and automating medical diagnosis and prediction [7,8].

Recent advances in technology have also drastically changed how people teach and learn. As Bonk et al [9] mentioned, “Recent technological developments have converged to dramatically alter conception of teaching and learning process.” These advancements offer innovative approaches to teaching and learning professionals within the life sciences field. For instance, Garcia-Pallares et al [10] and Tymcznska [11] discussed approaches in which instructors incorporate a course management system into life sciences professional training. In addition, Mantovani [12], Stansfield et al [13], and Barsom et al [14] addressed the potential to integrate virtual reality (VR) into training to enhance and improve learning experiences. As the rapid development and application of artificial intelligence (AI) continues [15], opportunities for adopting AI into training in the life sciences field will also become increasingly evident.

Although the fields of life sciences, teaching, and learning are moving forward, professional development of people in the life sciences field seems to fall behind. Gorman et al [2] pointed out that current and even future health care professionals are trained using the 100-year-old apprenticeship model, which is “see one, do one, and teach one.” In addition to the apprenticeship model, common strategies include lectures and films as the basis of a life sciences professional’s training, even though billions of dollars are dedicated to continuing educational activities in the life sciences worldwide [16,17]. When it comes to professional training in the life sciences, studies [18] indicate the similarities of the constant need for learning new knowledge, skills, and attitudes required due to the complexity of the field.

At presently, literature exploring the current state of teaching and learning in the life sciences field is sparse, especially within the professional training realm. Additionally, literature exploring the cost-effectiveness of these emerging approaches for training medical professionals is almost nonexistent. Although there is an underlying assumption that there is a direct relationship between continuing professional development and the performance of recipients, only a few studies have attempted to validate this assumption. According to Bloom et al, [19] and Umble and Cervero [20], continuing professional development can be effective, but its effectiveness varies.

In addition, a plethora of studies [21-24] have explored how emerging technologies can change the learning landscape across sectors. For instance, Dubey and Gunasekaran [25] investigated how AI can impact the transportation sector, and Gavish et al [26] explored how augmented reality (AR) and VR can transform industrial training. However, few studies have explored how emerging technologies can impact professional training and education in the life sciences field.

### Objectives

There is a limited amount of research into the educational tools and approaches currently employed within the life sciences. Furthermore, there is a lack of studies investigating how life sciences training might evolve under the influence of emerging technologies and the increasing emphasis on cost-effectiveness. This study aims to understand the current state of teaching and learning in the life sciences, and teaching professionals’ perceptions of the impact of new technologies and practices on the field. Specifically, this study will investigate the following:

- What technologies and pedagogies are educational professionals in Life Sciences Trainers & Educators Network (LTEN) member organizations using now?
- Which currently used approaches are most cost-effective and judged as most satisfactory by training and education professionals in the life sciences?
- How do life science training and education professionals think emerging technologies might change current practice in the near future?

## Methods

### Overview

This study used a mixed methods approach [27] to better understand the current state of teaching and learning in life sciences and training professionals’ perceptions of the impacts of emerging technologies. Mixed methods research requires data triangulation from quantitative and qualitative approaches, which strengthens the construct validity of the study [28]. In addition, 57 members from a life sciences education not-for-profit organization, LTEN, participated in the survey, and 9 participants who responded to the survey were interviewed. In compliance with the Pennsylvania State University Institutional Review Board protocols, all participants signed the informed consent release form.

### Quantitative Method

The survey consists of 18 broad questions with 15 subquestions in five specific sectors of the life sciences field (sales, clinical, product-related, customer-related, and other). Questions included demographic information along with detailed questions on the use and perceptions of pedagogies and tools.

### Qualitative Method

The primary data were collected through semistructured interviews. Interviews were conducted by phone and lasted approximately 40 minutes, covering 18 questions designed to follow-up findings from the survey questions. The interview protocol was designed based on the theoretical framework proposed by Seidmen [29], which consists of three general genres: personal experiences of emerging technology, attitudes toward specific technologies, and future expectations of emerging technologies. Researcher memos also served as a secondary data source [30].

## Results

### Demographics

Survey respondents represented the diversity of the LTEN membership. Founded in 1971, LTEN has grown to more than 1900 individual members who work in pharmaceuticals, biotech, medical device, and diagnostic companies, and industry partners who support the life sciences training departments. Additionally, LTEN has members across noncommercial disciplines including clinical, manufacturing, compliance, regulatory, quality, and general practice training roles [31]. This study invited 326 active members who are directly involved in the training department of member organizations, to participate in the survey through email. A total of 57 participants completed the survey.

As shown in Table 1, of the 57 participants, 24 (42%) of the respondents were education/training directors, 15 (27%) were corporate executives, 11 (19%) were education/training managers, and 7 (13%) were training developers. The respondents also had diverse responsibilities within their organizations (Table 2), with 23 (40%) working at US commercial-only organizations and 15 (26%) working at an entirely global organization.

**Table 1:** Respondents’ role.

Role	Respondents, n (%)^a^
Education/training developer	7 (13)
Education/training manager	11 (19)
Education/training director	24 (42)
Corporate executive responsible for education and training	15 (27)

^a^Percentages may not add up to 100 due to rounding.

**Table 2:** Respondents’ responsibilities.

Responsibility	Respondents, n (%)
US commercial operations only	23 (40)
An entire US organization	10 (18)
Global organization, but commercial operations only	9 (16)
An entire global organization	15 (26)

### Quantitative Results

#### Current Pedagogies Identified

A set of survey questions asked what pedagogies respondents currently use as their teaching strategies. Once they indicated their pedagogies, the respondents were asked to rank their selected pedagogies in order of importance. Fourteen pedagogies were presented as choices in the survey, and a weighted vote methodology was used to compare the pedagogies most commonly used and those perceived to be most important. A weighted ranking was produced by assigning a rank of 14 points to the item identified as most important, a score of 13 to the second most important, and so forth. The process was repeated for each category of trainee and for each training topic category, and a sum was calculated within each category of trainee and topic. The number of all the ranked items in each topic was used to produce a weighted percentage of the approaches used, which was divided by the total of all scores.

As shown in Table 3, there were 4 pedagogies (instructor-led training, virtual instructor-led training, online readings, and role play activities) that captured about 55% of the weighted importance rankings, with the other 10 pedagogies comprising the other half.

**Table 3:** Respondent-ranked importance of pedagogies.

Pedagogies	Weighted percentages^a^
Instructor-led training	17
Virtual instructor-led training	10
Role play activities	10
Competency-based learning	9
Case studies	9
Simulations	8
Field-based activities	8
Online readings	8
Collaborative learning	7
Problem-based learning	6
Games	4
Online discussions	2
Project-based learning	2
Other (please indicate)	1

^a^Percentages may not add up to 100 due to rounding.

**Figure 1:**
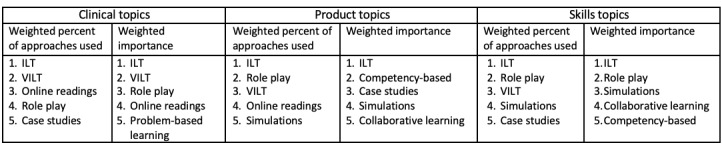
Weighted comparison between pedagogy use and importance in sales. ILT: instructor-led training; VILT: virtual instructor-led training.

Additionally, we identified some relationships in the differences between the pedagogies that respondents generally use and those they judged as most important. Figure 1 shows the top 5 ranked pedagogies in terms of use and importance (shown with numbers representing their ranks in each category) in sales across 3 training topics (clinical, product, and skills). Across all 3 topics, instructor-led training (ILT) is perceived to be the most used and most important pedagogy for salespeople, and virtual instructor-led training (VILT) methods are among the top 3 in terms of use but are much lower in terms of importance in product and skills topics.

#### Cost-effectiveness of Current Pedagogies Used

To determine respondents’ perceptions of the pedagogies and tools they use, we asked them to indicate a number between 0 and 100 that best indicated their satisfaction with each approach as well as its cost-effectiveness. To minimize the work and time required for respondents, we only asked them to do this for the top 3 pedagogies they had selected. We then averaged these satisfaction ratings and ranked approaches based on these averages. As shown in Table 4, the order of the satisfaction list indicates which approaches practitioners favor. There is a notable discrepancy between their satisfaction with a given approach and its cost-effectiveness.

In terms of satisfaction with the approaches, the most used approaches (ILT and VILT) are not the ones with which respondents are most satisfied. Project-based learning, the option with which most respondents were satisfied, was not among the most used.

In terms of cost-effectiveness, role-playing activities are most highly ranked, but are among the least frequently used. This may be due to the fact that role playing generally involves several trainees and trainers simultaneously, which may present logistical difficulties especially when conducted face-to-face. Interestingly, project-based learning, ranked as the most satisfying approach, is ranked as among the least cost-effective.

**Table 4:** Comparison between the most satisfying and most cost-effective approaches.

Rank	Most satisfying approach	Most cost-effective approach
1	Project-based learning	Role-play activities
2	Case study	Competency-based learning
3	Online discussion	Instructor-led training
4	Instructor-led training	Field-based learning
5	Problem-based learning	Problem-based learning
6	Other	Simulations
7	Competency-based learning	Other
8	Simulation	Online-reading
9	Virtual instructor	Project-based learning
10	Field-based learning	Case study
11	Collaborative learning	Virtual instructor
12	Role-play activities	Collaborative learning
13	Online reading	Games
14	Games	Online discussion

#### Now Versus the Future

We also asked respondents to consider a list of 8 technologies and asked how important they felt these technologies are now and how important respondents felt these technologies will be in 5 years, on a scale of 1-10. Respondents predicted a decrease in the importance of course or learning management systems (LMS) and a large increase in the importance of AI (Table 5). They also predicted that webinars and course development systems will decrease slightly in importance, while predicting that simulation creation tools will become the most important approach. A rather significant decline in the use of online games was also projected.

**Table 5:** Comparison between tools perceived to be most important now and in 5 years.

Rank	Now	Future
1	LMS^a^	Simulation creation tools
2	Webinars (live)	Artificial intelligence
3	Course development systems	Webinars (live)
4	Simulation creation tools	Course development systems
5	Online games	Virtual reality
6	Virtual reality	LMS
7	Augmented reality	Augmented reality
8	Artificial intelligence	Online games

^a^LMS: learning management systems.

### Qualitative Results

Qualitative phone interviews consisted of 18 questions related to contextual background and detailed information on both current learning strategies and experiences with technology. Participants’ perceptions of the future of training were also queried. After using an open coding approach [30] and thematic analysis [32] of 9 interview transcripts and researcher’s memos, the results revealed the emergence of three themes.

#### The Status Quo

All 9 respondents recognized that current training and education strategies in this field are at a clear risk of being abandoned in favor of rapidly evolving technologies. Most of the current techniques and tools have been used for some time, and the respondents noted that they are not very satisfied with them and that they do not perceive them to be very cost-effective. In particular, 8 of 9 respondents mentioned that the dominant learning tool, the LMS, is not conducive to learning, and the purpose of it is largely for administrative record tracking. One respondent said:

Most LMS have not developed with the user experiences in mind. In a global organizational level, LMS is just, “read the pdfs and do the quiz.” It’s absolutely boring.

Another respondent mentioned that LMS are generally not “mobile friendly,” adding that his sales team cannot have access to the learning material on-demand:

They are on the move all the time. It is unrealistic to expect them to have time to sit in the office and go through all the learning information.

Additionally, respondents raised concerns about measuring learning. Two respondents confessed that they have no valid understanding of whether learners are engaged with the tools and solutions currently employed and whether they are devoting the effort required to benefit in a meaningful way. One respondent said:

We only have a self-assessed checklist for our folks to fill out after the training session, but we do not know their progress at all.

Another respondent raised concern about how to devise valid evaluation metrics to assess his staff members. He provided an example that there are some staff members who got perfect scores on sales training assessments, yet have among the worst sales performance, while other staff members might have poor scores on sales training quizzes but have the highest sales performance in their district.

Respondents acknowledged the reality that making any change involving control and administration of the learning enterprise is challenging. However, respondents also pointed out that if properly leveraged, technologies offer vast opportunities for learning and could demonstrably enhance business productivity, even in the heavily regulated life science market where disruptive change is difficult to enact.

#### Call for Change

All the respondents identified that there is a clear call to action for change within both the life sciences learning professionals and the companies in which they work. More specifically, respondents indicated the need to provide new learning solutions and leverage technologies for both employees and customers. They reported the belief that this would help drive a successful business and that organizations not taking this approach would be at risk of being left behind. One respondent even joked that she did not want to be “asleep at the wheel.”

Throughout the interviews, all the respondents were reflective about their roles and performance as learning leaders and practitioners, coupled with a strong desire to be strategic in their decision-making concerning technology and to be open to change. One participant mentioned:

We, as practitioners and leaders alike, face a compelling need to improve in terms of competency, speed, quality, cost and overall return on investment.

Incorporating new technologies is perceived by these professionals as offering great promise, despite their awareness of the challenges that they realize will inevitably arise as they attempt to change the perceptions of those at the highest levels of their organizations toward embracing new solutions that leverage technology.

Additionally, respondents addressed the need for change to meet the training requirements for different generations and different ways of learning. One respondent said:

The old ways of teaching and training will not work on the young generations. Millennials are on their smartphones all the time; they are addicted to the technologies.

Other respondents indicated that people come from different backgrounds in learning and have different learning styles; therefore, the traditional ways of learning will become obsolete.

#### The Future of Teaching and Learning

When asked about the future and the roles technology might play in learning, all of the respondents described a future in which training programs and processes are accelerated, impactful, and engaging. One respondent said:

Our goal is to create learning experiences, so that when people walk away, they are like, wow, that never happened to me before, I am going to remember that. We are now integrating technologies to involve all the senses to create a new unique learning events to improve impacts and effectiveness.

They also mentioned seeing solutions evolve in which learners are given more control over the learning, and where learning practitioners shift from content developers to content curators. These predictions were based on observable trends, encompassing where technologies seem to be heading as well as the changing behaviors and product lines from the providers of learning materials and training development tools.

Additionally, respondents identified the potential to increase their impact through enhanced ability to develop social connectivity during learning, and to make learning personally relevant, interesting, engaging, easily accessible, self-driven, and even fun. Expanding on the concept of social connectivity, many commented on the growing nature of learner-centered environments in which learners engage interactive resources to meet their needs, working “on *their* terms” through learning experiences increasingly embedded in the workflow rather than as a separate formal learning event. One respondent said:

Learning is socio-cultural, if you limit the level of interactivity, you limit learning. We always need to look for opportunities for the learners to take control of the ability to connect and learn from each other. We need to look for ways to democratize data and have learning occurs [sic] down to the peer to peer level.

All respondents perceived AI, AR, and VR as having transformative near-term potential. One respondent commented that these emerging technologies are the ones “to take people’s knowledge and skills to the next level.” At the same time, two respondents indicated concern regarding how to adopt AI in this heavily regulated field.

## Discussion

### Principal Findings

This study explored the current state of training in the life science professional field through a mixed methods approach. Survey results indicated that instructor-led training is perceived to be the most used and most important pedagogy, and virtual instructor-led methods are among the top 3 in terms of use but are much lower in importance. In terms of satisfaction with the approaches, it is interesting to note that the most used approaches (instructor-led training and virtual instructor-led training) are not the approaches with which respondents were most satisfied. Although they were most satisfied with project-based learning, this approach was among the least cost-effective ones.

From the cost-effectiveness perspective, role-playing activities are most highly ranked, but are among the least frequently used. This may be due to the fact that role-playing generally involves several trainees and trainers simultaneously, which may present logistical difficulties, especially when conducted face-to-face, which may cause busy professionals to dislike the activity.

Looking into the future, both survey and interview results indicated that respondents are not satisfied with the status quo and that teaching and learning in this field need to change. Most of the techniques and tools currently used have been used for a long time, and the professionals are not very satisfied with them and do not find them very cost-effective. Unfortunately, the market and organizations in which these practitioners work are very complex, making change difficult. The interview results indicate that there is an evolution underway, but also highlight the need to get better at incorporating new technologies. This will require changing the perception that training and education are large expenses incurred by the organization without much evidence-based justification regarding effectiveness, and will require design thinking [33] to consider both new approaches and new ways to demonstrate the effectiveness of training efforts based on the contributions training and education make to the providing organizations. Interviewees described a future in which training programs and processes are accelerated, impactful, and engaging, and in which learners are given more control. Emerging technologies such as AI and VR were seen to have increasingly important roles to play, allowing learning to become more interesting, engaging, and perhaps even fun. The vision is of learner-centered environments in which learners engage interactive resources to meet their needs, perhaps in learning experiences that are increasingly embedded in the workflow. These professionals see a bright, exciting future and a challenging path to realize this vision.

### Limitations

Two limitations should be considered when interpreting these results. In the quantitative component of the study, 57 participants responded to the survey, which represents 17% of the sample pool of 326 people. However, this result is consistent with a typical noncompensated survey response rate (10%-15%) [34]. Future studies should expand the survey to a larger membership body to increase the number of respondents. In addition, the participants were recruited through a single organization (LTEN); future studies should expand recruitment to encompass a broader spectrum of education and training professionals in the life sciences.

### Conclusions

This is the first study in a series designed to better understand education and training in the life sciences on a macro level, in order to build a foundation for progress and evolution of the future landscape. All respondents in this study seemed very aware that rapid and potentially beneficial change is underway, fueled by emerging technologies. They acknowledge that while the pace of its emergence is increasing in less complex contexts, aspects of this particular industry seem likely to inhibit the pace of change. In addition, respondents also acknowledge that the adoption of emerging technologies is impeded by the absence of data demonstrating a compelling return on investment, and insufficient time and resources. As important as this perspective is, understanding is a necessary but not sufficient first step. Next steps involve developing strategies for how to extend this vision throughout the individual organizations. Beyond this, we will need to determine how to expose the existing dissatisfaction with traditional, ineffective ways of operating, and create realistic “first steps” in the desired direction. Then, leaders in the field must gather data, make modifications, adjust, and document the effects. The process is not unlike the development of the organization’s core products, from research and design to operational practices. The process will be challenging, particularly in the highly regulated, relatively conservative life sciences market. This set of challenges might become less daunting through projects like this initial study, and the extended conversations it may generate among those ready to act as pioneers.

## Abbreviations

AI: artificial intelligence
AR: augmented reality
ILT: instructor-led training
LMS: learning management systems
LTEN: Life Sciences Trainers & Educators Network
VILT: virtual instructor-led training
VR: virtual reality
